# LIN‐24 as a Molecular Switch: Dual Cytotoxic and Cytoprotective Roles of an Aerolysin‐Like Protein in *C. elegans*

**DOI:** 10.1002/jat.70053

**Published:** 2025-12-29

**Authors:** Sharoen Yu Ming Lim

**Affiliations:** ^1^ Department of Basic Medical Sciences, Faculty of Medicine and Health Sciences Universiti Malaysia Sarawak Kota Samarahan Sarawak Malaysia

**Keywords:** af‐PFP, cell‐death, donut‐shape mitochondria, LIN‐24, lipid metabolism

## Abstract

LIN‐24, an aerolysin‐like pore‐forming protein in *Caenorhabditis elegans* , exemplifies how ancient cytolytic mechanisms have evolved into regulated cellular processes. Initially identified for inducing nonapoptotic, engulfment‐dependent cell death in vulval precursor cells, LIN‐24 has emerged as a multifunctional regulator of metabolism, stress resilience, and immune defense. Its expression increases during starvation and bacterial infection, promoting lipid mobilization, mitochondrial remodeling, and activation of DAF‐16, MAPK, and SKN‐1 pathways, thereby enhancing survival and pathogen resistance. Conversely, gain‐of‐function mutations trigger cytotoxic membrane disruption, illustrating LIN‐24's dual role in cytotoxicity and cytoprotection. Despite these advances, its precise structure, regulatory mechanisms, and interaction networks remain undefined. Understanding how LIN‐24's pore‐forming activity is contextually controlled will clarify how eukaryotes repurpose toxic domains for adaptive functions and may provide translational insights into human pore‐forming proteins such as perforins and gasdermins involved in immune defense and programmed cell death.

## Abstract 2

LIN‐24 is an aerolysin‐like pore‐forming protein in *Caenorhabditis elegans* that illustrates how ancient cytolytic toxins are repurposed for adaptive cellular functions. Beyond inducing nonapoptotic cell death, LIN‐24 regulates metabolism, stress resistance, and immunity during starvation and infection by activating DAF‐16, MAPK, and SKN‐1 pathways. Its tightly controlled activity balances cytoprotection and cytotoxicity, offering insight into the evolution and regulation of eukaryotic pore‐forming proteins.

## 1 Introduction

Research on LIN‐24 is crucial because it provides fundamental insight into how cells balance survival and death through finely tuned pore‐forming mechanisms. As an aerolysin‐like pore‐forming protein (PFP) in *Caenorhabditis elegans* , LIN‐24 serves as an important model for understanding how eukaryotic organisms have adapted ancient cytolytic mechanisms for diverse physiological functions. Unlike classical bacterial PFPs that cause uncontrolled membrane disruption and cell lysis (Kulma and Anderluh 2021), LIN‐24 appears to act in a context‐dependent manner capable of inducing cell death under pathological conditions while contributing to stress resilience and tissue remodeling under physiological states. This functional duality highlights LIN‐24 as a unique molecular switch between cytotoxicity and cytoprotection, making it a valuable target for studying the evolutionary versatility of pore‐forming proteins. PFPs are a conserved class of molecules that form transmembrane pores to mediate immune defense, intercellular communication, or developmental regulation (Margheritis et al. 2023). In bacteria, aerolysin and its homologues are potent toxins that disrupt host cell integrity (Li et al. 2021). In eukaryotes including humans and *C. elegans* , however, pore‐forming proteins have been co‐opted for regulated processes such as apoptosis, autophagy, and immune activation (Galvin et al. 2008; Lan et al. 2025; Zhang et al. 2023).

Despite growing interest in LIN‐24, our understanding of its molecular mechanisms remains incomplete. Most studies rely on genetic mutants and transcriptomic analyses, which have revealed its involvement in processes such as mitochondrial remodeling, lipid metabolism, and innate immune signaling (Lan et al. 2025). However, the precise biochemical mechanisms underlying these functions are still unclear. No experimentally resolved three‐dimensional structure exists for LIN‐24, and the protein's oligomerization dynamics, membrane insertion behavior, and interaction with other cellular components remain uncharacterized. Furthermore, its expression patterns across developmental stages and tissue types are poorly defined, limiting our comprehension of its physiological roles.

Given these gaps, advancing research on LIN‐24 is essential for uncovering how aerolysin‐like proteins have been evolutionarily repurposed from toxic agents to regulated cellular modulators. A deeper understanding of LIN‐24 will not only clarify its role in *C. elegans* physiology but may also provide broader insight into the molecular evolution, regulation, and functional diversity of pore‐forming proteins in higher organisms. This review therefore aims to summarize current knowledge on LIN‐24, highlight its emerging roles in cellular homeostasis and defense, discuss structural and mechanistic limitations, and propose future research directions to address existing challenges.

## 2 Literature Search Strategy

A comprehensive literature search was conducted to identify studies relevant to LIN‐24, aerolysin‐like pore‐forming proteins, and their roles in cell death, stress adaptation, and *C. elegans* physiology. Searches were performed between Nov—Dec 2025 using PubMed, Google Scholar, Web of Science, and WormBase. Keywords and combinations included “LIN‐24”, “aerolysin‐like protein”, “pore‐forming protein”, “ *C. elegans* cell death”, “nonapoptotic cell death”, “engulfment pathways”, “stress resistance”, “cytoprotection”, and “starvation response”. Reference lists of key publications were also screened to identify additional sources. Both primary research articles and relevant reviews were included without date restrictions, although priority was given to peer‐reviewed studies with mechanistic or genetic insights. Non‐English articles, conference abstracts without full text, and papers lacking direct relevance to LIN‐24 or related pore‐forming proteins were excluded. All included literature was evaluated for methodological quality and relevance to the thematic scope of this review.

## 3 Overview of LIN‐24

LIN‐24, located on chromosome IV of *C. elegans* , encodes a 271‐amino‐acid protein containing an aerolysin‐like pore‐forming domain (residues 68–193). Structural modeling identified a conserved aerolysin‐like fold comprising five β‐strands and a hydrophobic–hydrophilic insertion loop, a trademark feature of pore‐forming proteins (Szczesny et al. 2011). Fluorescence imaging and qPCR analyses demonstrated constitutive LIN‐24 expression throughout the *C. elegans* lifespan, predominantly in the intestinal tract, suggesting a fundamental physiological role (Zhang et al. 2023). Knockdown of LIN‐24 by RNAi reduced maximum lifespan without altering median survival, implying its contribution to long‐term homeostasis and host defense (Zhang et al. 2023).

According to Wormbase (https://www.wormbase.org/), the *lin‐24* gene is expressed in multiple structures, including the germ line, germline precursor cells, interfacial epithelial cells, neurons, and pharyngeal muscle cells, as revealed by microarray, tiling array, RNA‐seq, and single‐cell RNA‐seq analyses. Its expression is influenced by several genes, such as *hsf‐1*, *ppm‐1.D*, and *fog‐1*, according to findings from microarray, tiling array, RNA‐seq, and proteomic studies. Furthermore, *lin‐24* is affected by five chemicals, including zidovudine, bortezomib, and paraquat, as demonstrated by RNA‐seq and microarray data. *lin‐24* and *lin‐33* are involved early in vulval precursor cell (VPC) development. Dominant mutations at either of these loci cause some VPCs to die. Surviving VPCs can generate vulval lineages, become 3″ or generate neuronal cells (Sternberg 1990). A number of mutations, exemplified by *n300* (which is associated with a reciprocal translocation and has not been assigned a gene name), cause VPCs to fuse with *hyp7*, the large hypodermal syncytium enveloping the animal (Sternberg 1990).

## 4 Cytotoxic Functions of LIN‐24

### 4.1 Pn.P Cell Death and Nonapoptotic Cytotoxicity

*C. elegans* lacks specialized “specific” phagocytic cells (Wang et al. 2024). Dead cell debris is typically phagocytosed by neighboring tissues, usually the epidermis (Wang et al. 2024). Phagocytosis of apoptotic cell debris by the epidermis can be observed during epidermal enclosure, indicating that the epidermis expresses phagocytic mechanisms during early development (Wang et al. 2024). In *C. elegans* , the engulfment and elimination of dying cells are not mediated by professional phagocytes; instead, they are performed by neighboring cells, which can be hypodermal, muscle, intestinal, and gonadal sheath cells (Lukácsi et al. 2021). One contains the phagocytic receptor CED‐1 (human SCARF‐1), the adaptor CED‐6 (GULP), the ABC transporter CED‐7 (ABCA), and the large GTPase DYN‐1 (Dynamin2). Two main, partly overlapping and conserved signaling pathways control the engulfment of apoptotic cells in *C. elegans* : the second path entails the CED‐2 (CrkII), CED‐5 (Dock180), CED‐10 (Rac1), CED‐12 (ELMO) proteins, the latter being the counterpart of the human *Rac* signaling. These two pathways partly converge at CED‐10 involved in actin polymerization, regulating the required cytoskeleton rearrangement for engulfment (Lukácsi et al. 2021).

Semi‐dominant gain‐of‐function mutations in the *C. elegans* genes *lin‐24* and *lin‐33* cause a unique form of nonapoptotic, cytotoxic cell death that selectively affects the vulval precursor (Pn.p) hypodermal cells. These mutations result in abnormal degeneration of Pn.p cells, producing a Vulvaless and egg‐laying defective (Vul and Egl) phenotype (Ferguson and Horvitz 1985; Galvin et al. 2008). The deaths occur independently of the canonical apoptotic regulators *ced‐3* and *ced‐4* but require the engulfment genes *ced‐2*, *ced‐5*, and *ced‐10* (Driscoll 1992; Ferguson and Horvitz 1985; Galvin et al. 2008). Blocking engulfment pathways can rescue the Pn.p cells from death, indicating that they are metabolically compromised but not fully dead, and that their premature removal by neighboring cells effectively being “eaten alive” results from inappropriate activation of the engulfment machinery (Ferguson and Horvitz 1985; Gumienny and Hengartner 2001). Dying Pn.p cells in *lin‐24* and *lin‐33* mutants exhibit abnormal morphologies when observed under Nomarski optics, consistent with ced‐3– and ced‐4–independent degeneration (Blum et al. 2008). These pathological cell deaths differ from classical apoptotic and necrotic processes, representing a distinct “cytotoxic” form of death (Galvin et al. 2008).

The semi‐dominant *lin‐24*(n432) and *lin‐24*(n1057) alleles cause nonapoptotic, phagocytosis‐dependent Pn.p cell death. The n1057 allele is an amber mutation suppressible by *sup‐5*, confirming its molecular identity (Ferguson and Horvitz 1985). These toxic effects represent gain‐of‐function rather than loss‐of‐function mutations, as the abnormal phenotype results from the neomorphic activity of the mutant LIN‐24 protein. Developmental conditions, such as passage through the dauer stage, can modulate the Vul phenotype (Ferguson and Horvitz 1985).

### 4.2 Engulfment Machinery and Genetic Interactions

Genetic studies have revealed that *ced‐2*, *ced‐5*, and *ced‐10* are necessary for *lin‐24*– and *lin‐33*–induced deaths, suggesting that these genes act not only in the engulfment of apoptotic cells but also in more general forms of cell elimination (Driscoll 1992). In contrast, *ced‐1*, *ced‐6*, *ced‐7*, and *ced‐8* may function primarily in programmed cell death. Indeed, *C. elegans* possesses two partially redundant engulfment pathways: one involving *ced‐2*, *ced‐5*, and *ced‐10*, and another composed of *ced‐1*, *ced‐6*, *ced‐7*, and *ced‐8*. Double mutants combining genes from each pathway exhibit strong defects in corpse clearance, whereas triple mutants show no further increase in severity, confirming two parallel but overlapping engulfment processes (Ellis et al. 1991). Either pathway can independently mediate engulfment, but when both are disrupted, unengulfed corpses persist or degrade through lysis and detachment (Ellis et al. 1991).

Pathological cell death of ventral epidermal blast cells in *lin‐24* and *lin‐33* mutants has been described as a form of cytotoxic death that does not resemble apoptosis or necrosis (Galvin et al. 2008). These deaths occur independently of *ced‐3* but are partially dependent on the phagocytic machinery (Joshi and Eisenmann 2004). In comparison, *pvl‐5* mutants exhibit *ced‐3–*dependent but *ced‐4–*independent nonapoptotic cell death, suggesting that epidermal cells may lack certain apoptotic components and thus rely on transcriptional activation of *ced‐3* to initiate death (Chisholm and Xu 2012; Joshi and Eisenmann 2004).

### 4.3 Aerolysin‐Like Mechanisms and Toxic Membrane Disruption

At the molecular level, *lin‐24* encodes an aerolysin‐like protein (ALP) homologous to bacterial pore‐forming toxins (Guo et al. 2019), while *lin‐33* encodes a novel protein of unknown structure or function (Galvin et al. 2008). The neomorphic (toxic gain‐of‐function) alleles of *lin‐24* and *lin‐33* likely disrupt cellular membranes and activate engulfment‐mediated cytotoxicity (Vlachos and Tavernarakis 2010). Together, *lin‐24* and *lin‐33* define a novel engulfment‐dependent, nonapoptotic cell death pathway in *C. elegans* that illustrates how aberrant activation of phagocytosis can directly mediate pathological cell elimination (Driscoll 1992; Ferguson and Horvitz 1985; Galvin et al. 2008; Vlachos and Tavernarakis 2010).

## 5 Cytoprotective Functions of LIN‐24

### 5.1 Starvation‐Induced Metabolic Reprogramming

Starvation induces a marked increase in *lin‐24* expression in *C. elegans* . Quantitative PCR and transcriptomic analyses confirm that *lin‐24* mRNA levels are significantly elevated after 24 h of food deprivation. Gene correlation analysis revealed that *lin‐24* expression during starvation is positively correlated with lipid metabolism‐related genes such as F38E9.1 (lipid metabolic process), Y46H3A.5 (lipid metabolic process), and *fat‐3* (biosynthesis of unsaturated fatty acids) (Lan et al. 2025). These associations suggest that LIN‐24 may coordinate lipid metabolism to provide alternative energy sources during starvation.

Overexpression of LIN‐24 (LIN‐24‐OE) further enhances this response, leading to the upregulation of 485 genes and downregulation of 119 genes, particularly those related to fatty acid metabolism and energy balance. KEGG pathway analysis identified significant enrichment in metabolic and lipid degradation pathways. Among the upregulated genes, *lipl‐3*, encoding a lipase enzyme, was notably elevated (Lan et al. 2025). LIPL‐3 catalyzes the breakdown of stored triglycerides into free fatty acids, which serve as critical energy substrates under nutrient scarcity. Supporting this, Oil Red O staining and LC–MS/MS analyses revealed that LIN‐24‐OE worms exhibit reduced lipid accumulation and enhanced lipid mobilization after 24 h of starvation compared to wild‐type (N2) controls (Lan et al. 2025). A summary of key genes regulated by LIN‐24 under starvation and stress conditions is provided in Table 1.

**TABLE 1:** Key gene and pathway connections.

Functional role	Associated genes/pathways	Evidence
Cytotoxic Pn.p cell death	*ced‐2*, *ced‐5*, *ced‐10* (engulfment); *lin‐33*	(Driscoll 1992; Ferguson and Horvitz 1985; Galvin et al. 2008)
Apoptotic clearance (contrast)	*ced‐1*, *ced‐6*, *ced‐7*, *ced‐8*	(Ellis et al. 1991)
Starvation lipid metabolism	*lipl‐3*, *fat‐3*, *F38E9.1*, *Y46H3A.5*	(Lan et al. 2025)
Mitochondrial remodeling	*mff‐1*, *mff‐2*, *drp‐1*, *clk‐1*	(Lan et al. 2025)
Immune defense	*daf‐16*, *pmk‐1*, *skn‐1*, *dod‐22*, *irg‐1*, *gst‐4*, *lys‐1*	(Zhang et al. 2023)
Developmental modulation	*lin‐33*, *let‐23*, *lin‐2*, *lin‐7*, *lin‐3*	(Moghal et al. 2003)

### 5.2 Starvation Resistance and Survival

Functional assays clearly demonstrate the protective role of LIN‐24 during starvation. Survival analysis shows that LIN‐24‐OE worms exhibit significantly higher survival rates under starvation conditions, while RNAi‐mediated knockdown of lin‐24 in both wild‐type and LIN‐24‐OE worms reduces survival, particularly after 24 h of food deprivation (Lan et al. 2025). Transcriptomic overlaps between LIN‐24‐OE and starved N2 worms confirm that LIN‐24 drives a transcriptional program similar to the natural starvation response, strengthening its role as a key metabolic regulator. The severity of the vulvaless phenotypes of certain *let‐23*, *lin‐2*, *lin‐7*, *lin‐3, lin‐24*, and *lin‐33* alleles is reduced by starvation and exit from dauer (Moghal et al. 2003).

### 5.3 Amino Acid Metabolism and Preservation of Muscle Integrity

In addition to its effects on lipid metabolism, LIN‐24 contributes to the maintenance of skeletal muscle structure during starvation. In N2 worms, nutrient deprivation leads to severe muscle degradation, including disrupted sarcomeres, thinner muscle fibers, and a loss of structural integrity (Lan et al. 2025). In contrast, LIN‐24‐OE worms maintain organized sarcomeres and intact muscle fiber architecture, even after 24 h of starvation (Lan et al. 2025). This preservation of muscle morphology suggests that LIN‐24 modulates amino acid metabolism and prevents excessive proteolysis, thereby conserving muscle proteins and sustaining locomotor function during energy stress. By stabilizing muscle structure, LIN‐24 contributes to long‐term starvation resistance and physical resilience in nutrient‐limited environments.

### 5.4 Mitochondrial Remodeling and Energy Conservation

Mitochondria are central to energy metabolism, and their dynamic remodeling is critical for survival during starvation. Transcriptomic analyses indicate that LIN‐24 negatively regulates ATPase activity, implying a shift toward energy conservation. Electron microscopy revealed a striking increase in donut‐shaped mitochondria in LIN‐24‐OE worms after 24 h of starvation (Lan et al. 2025). Donut‐shaped mitochondria characterized by ring‐like morphologies are thought to reduce mitochondrial surface area, decrease ATP production, and minimize energy expenditure. This structural adaptation allows cells to conserve energy during nutrient deprivation.

To elucidate the genetic basis of this phenomenon, RNAi knockdown of *mff‐1*, *mff‐2*, *drp‐1*, and *clk‐1* genes essential for mitochondrial fission and remodeling was performed (Lan et al. 2025). The suppression of these genes significantly reduced survival in LIN‐24‐OE worms, confirming that LIN‐24‐mediated mitochondrial remodeling depends on this mitochondrial dynamics network. Seahorse metabolic assays further demonstrated reduced basal respiration rates in LIN‐24‐OE worms, both under normal and starved conditions, reinforcing the link between mitochondrial morphology and energy economy (Lan et al. 2025). In humans, mitochondria undergo distinct shape changes during starvation, either swelling or forming donut‐like structures (Zhou et al. 2020). Donut‐shaped mitochondria are especially resistant to autophagy: even after depolarization, they do not recruit the autophagy receptors CALCOCO2 and OPTN because PRKN‐mediated ubiquitination does not advance to the steps required for receptor engagement (Zhou et al. 2020).

### 5.5 Immune Induction During Infection

Upon exposure to *Pseudomonas aeruginosa* PA14, LIN‐24 mRNA expression increased significantly (Zhang et al. 2023). RNAi‐mediated LIN‐24 knockdown rendered worms highly susceptible to PA14 infection, shortening median survival from 132 to 84 h and increasing bacterial intestinal load. This indicates LIN‐24 is pathogen‐inducible and crucial for infection resistance.

Transgenic worms overexpressing LIN‐24 (LIN‐24‐OE:GFP) exhibited increased mRNA and protein levels of LIN‐24 and markedly improved survival under bacterial infection. LIN‐24‐OE worms showed longer survival after *P. aeruginosa* (132 vs. 96 h in controls), reduced bacterial load, and enhanced resistance to *Staphylococcus aureus* ATCC 6538 (120 vs. 96 h). Feeding LIN‐24‐OE worms with LIN‐24 RNAi bacteria abolished this protection, confirming that LIN‐24 directly mediates antimicrobial resistance (Zhang et al. 2023).

### 5.6 Activation of Immune Gene Networks and Stress Pathways

Weighted gene co‐expression network analysis (WGCNA) of LIN‐24‐OE worms identified 478 highly correlated genes, predominantly enriched in immune and defense responses (Zhang et al. 2023). Transcription factor analysis revealed strong correlations with ZBTB, zf‐C2H2, and forkhead family (DAF‐16) transcription factors (Zhang et al. 2023). Given DAF‐16's established role in *C. elegans* immunity, these findings suggest that LIN‐24 enhances immune defense through forkhead/DAF‐16 transcriptional activation.

LIN‐24 does not directly kill bacteria like CDC‐type PFPs (e.g., perforin‐2, complement C9) (Zhang et al. 2023). Instead, it activates host immune pathways. LIN‐24‐OE worms showed elevated expression of antimicrobial genes including dod‐22, irg‐1, T24B8.5, lys‐1, lys‐8, gst‐4, and K08D8.5 (Zhang et al. 2023). dod‐22 is a DAF‐16 downstream target, and DAF‐16 RNAi reduced LIN‐24‐mediated survival by 36.3% under PA14 infection. Knockdown of dod‐22 similarly abolished LIN‐24‐mediated protection. Disruption of pmk‐1 (MAPK) and skn‐1 (oxidative stress pathway) reduced survival, while dbl‐1 (TGF‐β pathway) had no effect. These results indicate that LIN‐24 enhances microbial resistance through DAF‐16‐, MAPK‐, and SKN‐1–dependent immune signaling, rather than direct bactericidal activity.

### 5.7 Integrated Mechanistic Model

**FIGURE 1:**
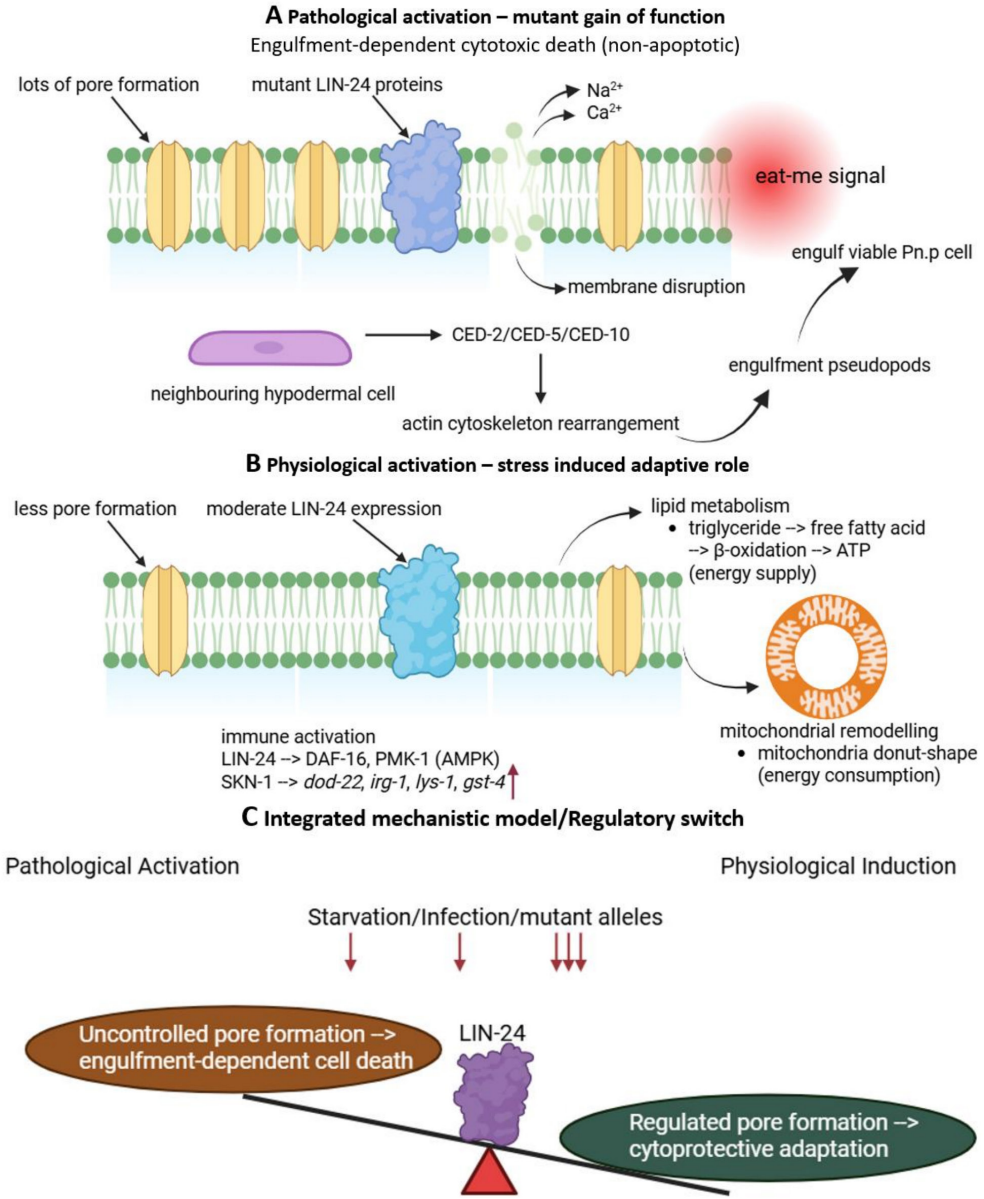
Dual functional and integrated model of LIN‐24 activity in *C. elegans* . (A) Under pathological gain‐of‐function mutations (*lin‐24*, *lin‐33*), excessive pore formation disrupts Pn.p cell membranes, exposing “eat‐me” signals that activate the CED‐2/CED‐5/CED‐10 engulfment pathway, leading to nonapoptotic cytotoxic death. (B) Under physiological induction by starvation or bacterial infection, moderate LIN‐24 activation promotes lipid catabolism, mitochondrial remodeling, and immune gene expression via DAF‐16, PMK‐1, and SKN‐1, enhancing stress resilience and survival. (C) Integrated model: the biological outcome of LIN‐24 activity depends on its activation level and cellular context, functioning as a molecular switch between cytotoxic and cytoprotective states.

Collectively, available evidence supports a unified model in which LIN‐24 acts as a bifunctional pore‐forming effector whose outcome depends on expression level, cellular context, and genetic background (Figure 1A–C). Thus, LIN‐24 exemplifies how a pore‐forming protein, depending on regulatory control, can act as either a pathological effector of cell death or a vital coordinator of stress resilience and immune defense in *C. elegans* .

## 6 Conclusion and Future Perspectives

In summary, LIN‐24 represents a remarkable example of how ancient pore‐forming mechanisms have been evolutionarily adapted for complex physiological regulation. Originally characterized for its role in cell death and developmental arrest in *C. elegans* , LIN‐24 is now recognized as a multifunctional protein implicated in cellular homeostasis, stress resilience, and immune defense. Its ability to shift between cytotoxic and protective roles underscores a sophisticated regulatory network that fine‐tunes pore‐forming activity according to cellular context. This functional versatility positions LIN‐24 as a valuable model for exploring how organisms repurpose potentially harmful molecular structures for beneficial outcomes.

However, current knowledge of LIN‐24 remains incomplete. Most studies rely on genetic and transcriptomic analyses, which provide limited insight into the protein's biochemical properties and regulatory dynamics. The absence of an experimentally resolved structure leaves many questions regarding its oligomerization state, pore formation, and membrane interaction unanswered. Furthermore, its subcellular localization, trafficking patterns, and precise molecular partners have not yet been clearly identified. These gaps hinder our understanding of how LIN‐24 integrates into broader signaling networks governing metabolism, mitochondrial function, and innate immunity.

Future research should therefore employ multidisciplinary approaches to elucidate LIN‐24's structure and function at multiple biological levels. High‐resolution structural studies using cryo‐electron microscopy or X‐ray crystallography are needed to confirm its predicted aerolysin‐like β‐pore architecture and to visualize conformational transitions during activation. Proteomic and interactome mapping could identify binding partners and posttranslational modifications that regulate its activity. Meanwhile, single‐cell transcriptomics, spatial imaging, and tissue‐specific genetic models would help clarify how LIN‐24 expression and function vary across developmental stages and physiological states. Integrating these approaches will provide a more comprehensive picture of LIN‐24's biological roles and its regulation under different stress conditions.

Beyond *C. elegans* , the study of LIN‐24 carries important translational implications for human biology. Many human proteins, such as perforins, complement components, and gasdermins, share structural and functional similarities with LIN‐24, mediating pore formation during immune defense, inflammation, and programmed cell death. Understanding how LIN‐24 balances its cytolytic and regulatory roles may shed light on the conserved mechanisms that control membrane integrity and cellular resilience in mammals. Ultimately, continued exploration of LIN‐24 promises not only to unravel fundamental aspects of pore‐forming protein biology but also to inspire novel strategies for modulating cell survival, stress tolerance, and immune function in human health and disease.

## Author Contributions

**Sharoen Yu Ming Lim:** conceptualization, data curation, formal analysis, visualization, writing – original draft, writing – review and editing.

## Conflicts of Interest

The author declares no conflicts of interest.

## Data Availability

Data sharing not applicable to this article as no datasets were generated or analysed during the current study.
